# Menthol-Induced Chirality in Semiconductor Nanostructures: Chiroptical Properties of Atomically Thin 2D CdSe Nanoplatelets Capped with Enantiomeric L-(−)/D-(+)-Menthyl Thioglycolates

**DOI:** 10.3390/nano14231921

**Published:** 2024-11-28

**Authors:** Maria Yu. Skrypnik, Daria A. Kurtina, Sofia P. Karamysheva, Evgeniia A. Stepanidenko, Irina S. Vasil’eva, Shuai Chang, Alexander I. Lebedev, Roman B. Vasiliev

**Affiliations:** 1 Department of Chemistry, Lomonosov Moscow State University, 119991 Moscow, Russia; skrypnikmy@my.msu.ru (M.Y.S.); dashutakarlova@mail.ru (D.A.K.); 2 PhysNano Department, ITMO University, Kronverksky pr.49, 197101 St. Petersburg, Russia; spkaramysheva@itmo.ru (S.P.K.); eastepanidenko@itmo.ru (E.A.S.); 3 A. N. Bach Institute of Biochemistry, Research Center of Biotechnology of the Russian Academy of Sciences, Leninsky Ave. 33, Bld. 2, 119071 Moscow, Russia; ir-vas@yandex.ru; 4 Faculty of Materials Science, Shenzhen MSU-BIT University, Shenzhen 518115, China; schang@smbu.edu.cn; 5 Department of Physics, Lomonosov Moscow State University, 119991 Moscow, Russia; swan@scon155.phys.msu.ru; 6 Department of Materials Science, Lomonosov Moscow State University, 119991 Moscow, Russia

**Keywords:** 2D semiconductors, chirality, CdSe nanoplatelets, circular dichroism, menthol, excitons, ligand exchange, colloidal synthesis

## Abstract

Semiconductor colloidal nanostructures capped with chiral organic molecules are a research hotspot due to their wide range of important implications for photonic and spintronic applications. However, to date, the study of chiral ligands has been limited almost exclusively to naturally occurring chiral amino and hydroxy acids, which typically contain only one stereocenter. Here, we show the pronounced induction of chirality in atomically thin CdSe nanoplatelets (NPLs) by capping them with enantiopure menthol derivatives as multi-stereocenter molecules. L-(−)/D-(+)-menthyl thioglycolate, easily synthesized from L-(−)/D-(+)-menthol, is attached to Cd-rich (001) basal planes of 2- and 3-monolayer (ML) CdSe NPLs. We show the appearance of narrow sign-alternating bands in the circular dichroism (CD) spectra of 2 ML NPLs corresponding to heavy-hole (HH) and light-hole (LH) excitons with maximal dissymmetry g-factor up to 2.5 × 10^−4^. The most intense CD bands correspond to the lower-energy HH exciton, and in comparison with the N-acetyl-L-Cysteine ligand, the CD bands for L-(−)-menthyl thioglycolate have the opposite sign. The CD measurements are complemented with magnetic CD measurements and first-principles modeling. The obtained results may be of interest for designing new chiral semiconductor nanostructures and improving understanding of their chiroptical properties.

## 1. Introduction

Chiral inorganic nanostructures are attracting tremendous interest in the fields of photonics and spintronics because they strongly interact with circularly polarized light (CPL) by absorbing or emitting the left-handed or right-handed photons [1,2] and also demonstrate chiral-induced spin selectivity effects (CISS) in charge carrier transport [3,4]. Recently, significant progress has been made in the synthesis and application of chiral semiconductor nanostructures [5,6,7]. The manifestation of mirror asymmetry makes chiral semiconductor nanostructures candidates for a number of important applications, including chiral sensing, asymmetric catalysis, enantiomer separation, and as CPL emitters and detectors [8,9,10].

The origin of the chirality of nanostructures should be addressed to their twisted shape, an intrinsic chirality of the material, the chiral assembly of nanostructures, or the presence of chiral organic molecules (ligands) on their surface. In the latter case, the mechanism for the appearance of chirality is the chirality induction [1]. Enantiomers of organic molecules containing a stereocenter and an anchor group for attachment to the surface of the nanostructure are used to induce chirality. This design has been generally accepted since the first publication by Molony et al. [11], in which chiral cysteine molecules with an anchor sulfhydryl group were used to induce chirality in CdS nanoparticles. The most popular chiral ligands are thiol-containing molecules; for example, cysteine, penicillamine [12], acetylcysteine [13], and glutathione [14] are used. With cysteine-based ligands, the induction of chirality has been achieved in nanoparticles of different classes of inorganic semiconductors: from quantum dots, nanorods, and nanoplatelets (NPLs) of cadmium chalcogenides [15,16,17] to molybdenum disulfide and graphene quantum dots [18,19]. To describe the degree of chirality of nanostructures in their interaction with CPL, the dissymmetry factor g is used, which is calculated as g = ΔA/A = (A_L_ − A_R_)/A, where A_L_ and A_R_ are the absorbances of circularly polarized left-handed and right-handed light, respectively, and A is the absorbance of unpolarized light. The maximum dissymmetry factors achieved for thiolate ligands in the case of CdSe NPLs were 3 × 10^−3^ for acetylcysteine [20] and 6 × 10^−3^ for cysteine [21]. Thiol-free naturally occurring chiral amino and hydroxy acids, the attachment of which to the surface is achieved due to their carboxyl group, have also been used to induce chirality [22,23,24]. Chiral amines (R/S-methylbenzylamine, etc.) are used to induce chirality in the case of hybrid perovskites [25]. As a rule, all the chiral molecules considered above contain a single stereocenter. An exception to this rule is tartaric acid, whose derivatives contain two stereocenters and which was recently used to induce chirality in CdSe nanoplatelets with a dissymmetry factor of 2 × 10^−3^ [26]. Thus, the set of chiral ligands that can be used for the induction of chirality is quite limited and the search for new optically active ligands is of exceptional interest.

In this work, we selected menthol stereoisomers to develop a new chiral ligand for chirality induction in semiconductor nanostructures. Menthol has three stereocenters, of and its most well-known stereoisomers are the naturally occurring (1R, 2S, 5R) one called L-(−)-Menthol and its opposite (1S, 2R, 5S) stereoisomer called D-(+)-Menthol. The L-menthol enantiomer is a natural, readily available compound whose derivatives are widely used in asymmetric organic synthesis as chiral auxiliaries for asymmetric induction [27,28,29,30] and chiral phase-transfer catalysis [31], demonstrating high efficiency. Previously, to the best of our knowledge, the menthol derivatives have not been used for the induction of chirality in nanostructures despite their high optical activity and wide application in organic chemistry. We used colloidal CdSe nanoplatelets with zinc blendestructure as a representative of semiconductor quantum wells with a 2D electronic structure. Colloidal CdSe nanoplatelets allow achieving a high dissymmetry factor [20,21,32] and are, at the same time, interesting for practical applications such as LEDs, lasers, and white light LEDs [33,34,35]. The unique properties of such nanoplatelets are manifested in the dense packing and self-assembly of ligand molecules on their basal planes, which may be of interest for the construction of 2D membranes with permeable double-layers between the nanoplatelets [36,37]. We chose NPLs with extremely small thicknesses, for which the CdSe semiconductor core consists of 2 and 3 monolayers (where monolayer (ML) is a pair of atomic planes of Cd and Se atoms), each less than 1 nm, which ensures maximum exciton confinement. We show the pronounced induction of chirality in atomically thin CdSe NPLs by capping them with thioglycolic ester of L-(−)/D-(+)-menthol (L-(−)/D-(+)-menthyl thioglycolate or L-/D-menthylTG) as an enantiopure menthol derivative.

## 2. Materials and Methods

Cadmium acetate dihydrate (Cd(CH_3_COO)_2_∙2H_2_O, ≥98%), selenium powder (Se, 99.99%), trioctylphosphine (TOP, 90%), oleic acid (OA, 90%), 1-octadecene (ODE, 90%), L- and D-menthol (≥98%), thioglycolic acid (TGA, ≥98%), phosphoric acid (≥99%), tetrahydrofuran (THF, ≥99%), N-acetyl-L-cysteine (L-ACC, ≥99%), and solvents were purchased from Sigma-Aldrich, St. Louis, MO, USA.

### 2.1. Synthesis of the L-(−)/D-(+)-Menthyl Thioglycolate

In a 10 mL round-bottom flask, L- or D-Menthol (1.6 g, 10 mmol) was dissolved in 5 mL of toluene and 0.45 mL (10 mmol) of thioglycolic acid, and 100 μL H_3_PO_4_ as a catalyst was added. The mixture was heated at 100 °C for 24 h under argon flow then cooled to room temperature, after which the toluene was removed under reduced pressure. The mixture of unreacted menthol and desired ester was distilled under 4 mm Hg pressure and fraction was boiled at 90 °C to obtain the pure product.

Thioglycolic ester of L-(−)-Menthol (L-menthylTG) is a colorless liquid, and the yield was 80% (C_12_H_22_O_2_S, MW: 230 g/mol). 1H NMR (600 MHz, Chloroform-d) resulted in: δ 0.75 (d, 3H, CH_3_), 0.9 (d, J = 3.18 Hz, 3H, CH_3_), 0.92 (d, J = 4.22 Hz, 3H, CH_3_), 0.92–1.09 (m, 3H), 1.34–1.55 (m, 2H), 1.55–1.74 (m, 2H), 1.82–2.06 (m, 3H), 3.21 and 3.23 (d, J = 8.3 Hz, 2s, 2H, CH_2_S), and 4.70 (td, J = 10.9, 4.4 Hz, 1H, CHOCO).

### 2.2. Synthesis of 2 and 3 ML CdSe NPLs Capped with Oleic Acid

Synthesis of 2 and 3 ML CdSe NPLs capped with oleic acid was performed according to [20]. Briefly, 80 μL of oleic acid and 0.13 g of cadmium acetate dihydrate were placed into a flask containing 10 mL of ODE that was degassed prior to the synthesis. The reaction mixture was heated up to 120 °C for 2 ML CdSe NPLs or 180 °C for 3 ML CdSe NPLs under argon flow. Once the temperature was reached, 125 μL of 1 M solution of selenium in trioctylphosphine diluted up to 0.5 mL by ODE was injected rapidly into the reaction mixture under vigorous stirring. After that, growth proceeded for 6 h for 2 ML CdSe NPLs or 1 h for 3 ML CdSe NPLs. Finally, the reaction was quenched by rapid injection of 2 mL of OA and then the flask was cooled to room temperature. CdSe NPLs were precipitated by adding an equal volume of acetone, separated by centrifugation at 5000 rpm for 10 min, and washed several times with acetone. White (2 ML) or yellow (3 ML) precipitate was finally redispersed in 2 mL of hexane.

### 2.3. Ligand Exchange Synthesis of 2 and 3 ML CdSe NPLs Capped with Oleic Acid

Ligand exchange of as-grown 2 and 3 ML CdSe NPLs with chiral menthylTG was performed in aprotic THF solvent. Typically, 50 μL of menthylTG (L- or D-stereoisomer) was dissolved in 1 mL of THF and 200 μL of as-synthesized CdSe NPLs in THF was added. The mixture turned yellow to orange as ligand exchange started. After 24 h of reaction, 1 mL of acetone was added and exchanged NPLs were separated by centrifugation. Finally, CdSe NPLs covered with menthylTG were dissolved in 2 mL of methanol. Ligand exchange of as-grown 2 and 3 ML CdSe NPLs with TGA or L-ACC was carried out similarly [20,32].

### 2.4. Methods

Transmission electron microscopy (TEM) images were obtained on an LEO19 AB OMEGA microscope operated at 100 kV. Fourier-transform infrared spectroscopy (FTIR) spectra were registered on a Perkin-Elmer Frontier spectrometer in the 400–4000 cm^−1^ wavenumber range at room temperature. Samples for analysis were prepared by mixing a drop of NPL solution in tetrahydrofuran with KBr powder, followed by pressing into tablets after solvent evaporation. H-NMR spectroscopy was carried out on Agilent 400-MR equipment. UV-vis absorption spectroscopy was carried out in the 200–800 nm wavelength range with a scanning speed of 100 nm/min on a Carry50 (Varian) spectrophotometer. Circular dichroism (CD) spectra were recorded on Chirascan spectropolarimeter (Applied Photophysics, Leatherhead, UK) in the 200–500 nm wavelength range with a scanning speed of 10 nm/min, a 1 nm step, and an integration time of 3 s. All optical measurements were carried out at room temperature on colloidal solutions of NPLs in tetrahydrofuran or methanol diluted to an optical density of <1. Quartz cuvettes (Hellma Analytics, Müllheim, Germany) with 0.2 cm optical path length were used. Magnetic circular dichroism (MCD) spectra under magnetic field up to ±1.5 tesla were measured on J-1500 (Jasco, Tokyo, Japan) CD spectropolarimeter equipped with electromagnetic unit MCD-581.

The geometry of the (001)-oriented 2 ML CdSe nanoplatelet covered at both sides with L-menthylTG was calculated from first principles within the density functional theory using the ABINIT 7.9.4 software [38]. PAW pseudopotentials [39], the plane wave basis set with a cut-off energy of 30 Ha (816 eV), and the local density approximation were used. The molecule was placed in a cubic box with the 30 bohr sides. Six possible orientations of the TGA group with a fixed position of the bridging O atom against the menthol base were tested, and the lowest-energy structure was accepted as a molecular structure. The nanoplatelet was modeled using a long supercell containing a vacuum gap of 18 bohr; the integration over the Brillouin zone was performed on a 6 × 6 × 1 Monkhorst–Pack mesh. The relaxations of all structures was continued until the forces acting on the atoms became less than 5 × 10^−6^ Ha/bohr (0.25 meV/Å).

## 3. Results and Discussions

### 3.1. Synthesis of Chiral Ether and Ligand Exchange on the Surface of Nanoplatelets

**Figure 1:**
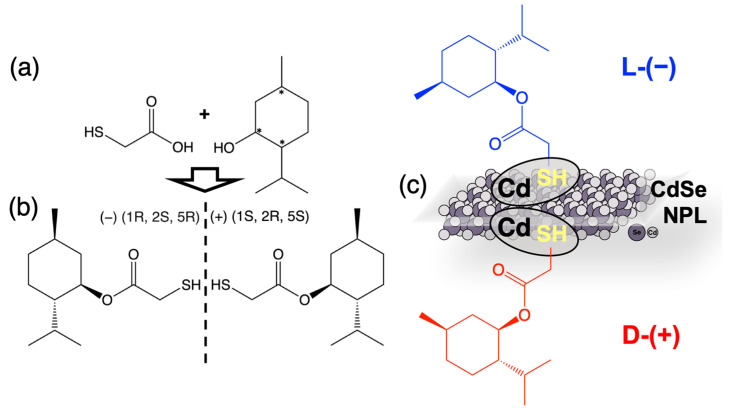
(**a**) Scheme of synthesis of chiral ester from menthol and thioglycolic acid. (**b**) Structural formulas of mirror stereoisomers of L-(−)/D-(+)-menthyl thioglycolate. (**c**) Scheme of attachment of chiral ester to basal planes of CdSe nanoplatelets due to formation of cadmium–sulfur bond. Note the nonpolar outer surface of the obtained nanoplatelets, caused by cyclohexane fragments of the ester.

We designed a chiral ligand L-(−)/D-(+)-menthyl thioglycolate by combining menthol and thioglycolic acid via an ester bond between the menthol hydroxyl group and the carboxyl group of thioglycolic acid. The sulfhydryl group of thioglycolic acid acts as an anchor coordinating the resulting ester on the basal cadmium planes of CdSe NPLs, and the menthol fragment containing three stereocenters induces chirality (Figure 1). The esterification reaction of the L/D enantiomers of menthol with thioglycolic acid proceeded readily at 100 °C in the presence of catalytic traces of phosphoric acid, with preservation of the enantiomeric purity of the resulting ester. The specific optical rotation [*α*] 589 for the L-(−)-menthyl thioglycolate was −76 ± 1°, which is higher than the value of −49 ± 1° used for pure L-(−)-menthol.

**Figure 2:**
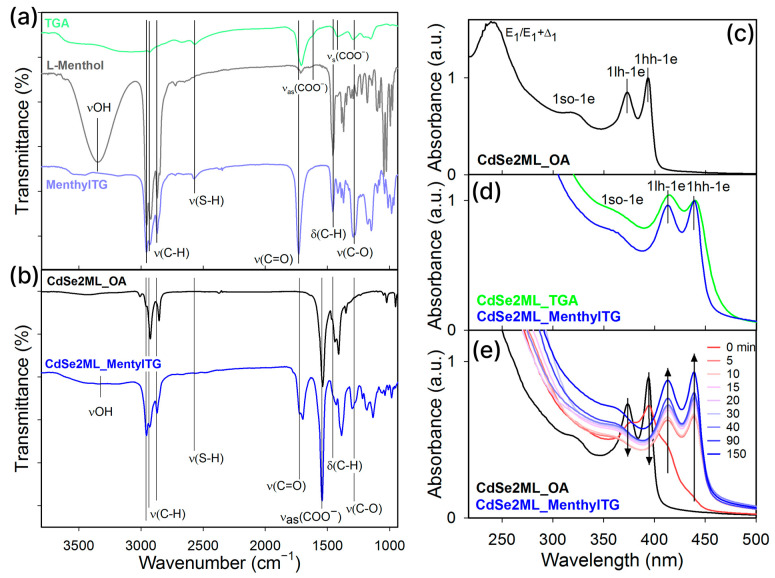
(**a**) FTIR spectra of the initial reagents, TGA (green line) and L-menthol (grey line), and spectrum of the synthesized ester menthylTG sample (blue line). (**b**) FTIR spectra of menthylTG-capped CdSe2ML_menthylTG (blue line) sample. Black line shows the FTIR spectrum of the initial OA-capped CdSe2ML_OA sample. Positions of the main vibration bands are marked by black vertical lines. The spectra are offset for clarity. Typical absorbance spectra of (**c**) CdSe2ML_OA NPLs (black solid line) and (**d**) the same sample after ligand exchange with menthylTG (CdSe2ML_menthylTG, blue line) and thioglycolic acid (CdSe2ML_TGA, green line) ligands. (**e**) Modification of absorbance spectra during the ligand exchange at different times. Black arrows show changes in the intensity of bands during the ligand exchange.

To evaluate the success of the esterification reaction, FTIR was used to analyze the initial reagents, TGA and L-menthol, as well as the synthesized menthylTG, which was also characterized by H-NMR (Figure S1 in the Supporting Information). A clear difference between the spectra of the menthol and TGA molecules (Figures S2 and S3) and the spectrum of menthylTG is visible, confirming the esterification reaction. The infrared spectra are shown in Figure 2a. In the spectrum of thioglycolic acid, we observed vibrations of the key functional groups: stretching vibrations of the sulfhydryl group (SH) at 2567 cm^−1^, stretching vibrations of C=O of the carboxylic group at 1724 cm^−1^, and small asymmetric (1581 cm^−1^) and symmetric (1405 cm^−1^) stretching vibrations for the ionized form of the acid [40,41,42]. Additionally, stretching vibrations in the range of 2800–3000 cm^−1^ for CH groups were also evident. The spectrum of L-menthol revealed intense stretching vibrations of the hydroxyl group at 3331 cm^−1^ and of CH groups in the range of 2800–3000 cm^−1^. Furthermore, bending vibrations of CH bonds at 1460 cm^−1^ were observed. For the synthesized menthylTG, there was a clear presence of intense vibrations from the CH groups of the cyclohexane ring, with the absence of any vibrations from the hydroxyl group. Simultaneously, there were vibrations of the sulfhydryl group of thioglycolic acid, as well as bending vibrations of the C=O of the carboxylic group and the newly formed C–O bonds. This leads us to the conclusion that the esterification process was successful.

The chiral ester was effectively cross-linked to the surfaces of CdSe NPLs. For this purpose, CdSe NPLs of 2 ML thickness with the lowest-energy HH exciton band of 394 nm (CdSe2ML_OA) and of 3 ML thickness with the lowest-energy HH exciton band of 463 nm (CdSe3ML_OA) were used. The sulfihydryl terminal group of the ester was attached to the cadmium basal surface, easily replacing the carboxyl group of the native oleic acid, which allowed us to obtain nanoplatelets that were completely covered with menthylTG ligands (CdSe2ML_menthylTG and CdSe3ML_menthylTG). Ligand exchange was conducted using the organic solvent method in tetrahydrofuran (THF) solvent, according to our earlier reported protocol [20]. The obtained NPLs had a non-polar surface due to the hydrocarbon cyclohexane fragment of menthol having an affinity for non-polar solvents, which ensured easy dissolution in THF and the formation of stable dispersions of menthylTG-coated NPLs. We proved the replacement of the OA ligand on the basal surfaces of NPLs by menthylTG ligands by FTIR (Figure 2b). For the initial nanoplatelets coated with oleic acid, a set of pronounced bands of negatively charged carboxylate and aliphatic carbon chain vibrations were observed, which is consistent with reference [20]. For the NPLs with the menthylTG, most of the vibration bands of the pure menthylTG ligands were detected together with disappearance the bands of negatively charged carboxylate and aliphatic carbon chain vibrations that are typical of the OA ligand. The disappearance of the vibrations of the sulfhydryl group suggests the binding of this group to the surface cadmium.

The optical properties of the initial OA-capped NPLs and the sample after ligand exchange with menthylTG were studied by UV-vis spectroscopy. The absorbance spectrum of CdSe2ML_OA (Figure 2c) demonstrates pronounced narrow exciton transitions involving the heavy-hole (HH) and light-hole (LH) bands. In the ultraviolet range, a high-energy E_1_/E_1_ + Δ_1_ exciton band related to transitions at the boundary of the Brillouin zone [43] was observed. After menthylTG ligand exchange, the absorbance spectra of the CdSe2ML_menthylTG NPLs demonstrated spectral shifts of both HH and LH exciton bands to a longer wavelength (Figure 2d and Figure S4), which was also the case for the E_1_/E_1_ + Δ_1_ exciton band. This indicates the addition of sulfur atoms from the sulfhydryl group to the surface cadmium atoms and the formation of a sulfide layer on the surface, thus causing an increase in the width of the potential well due to an increase in the thickness of the nanoplatelets, as previously reported in the literature for thiolate ligands [20,44]. To prove the role of the sulfhydryl group, we also synthesized a sample coated with thioglycolic acid, CdSe2ML_TGA (Figure 2d and Figure S4). A similar shift of about 40 nm was observed for both the TGA and menthylTG ligands, which shows contribution from the sulfur layer. The spectra’s evolution during the exchange of the OA ligand for the menthylTG ligand is shown in Figure 2e. The addition of menthylTG is easily seen by the appearance of exciton bands shifted to the long-wavelength spectral region, while the initial exciton bands disappear, as shown by the arrows. It is necessary to note the rapidity of the exchange: after 20 min at room temperature, only the lines with the maximum spectral shift were observed in the spectra. At the same time, the exchange retained narrow exciton bands.

**Figure 3:**
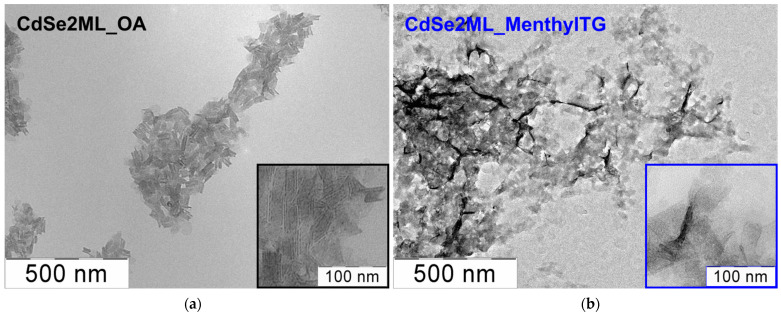
TEM images of the transformation of as-synthesized scroll-like CdSe2ML_OA NPLs covered with oleic acid ligands (**a**) to flat CdSe2ML_menthylTG NPLs after ligand exchange with L-menthylTG (**b**). Insets: enlarged images of NPL ensembles.

The analysis of the nanoplatelet morphology changes after the ligand exchange was conducted using TEM. Figure 3 shows TEM images of the as-synthesized CdSe2ML_OA NPLs coated with oleic acid ligands and the same NPLs after ligand exchange with menthylTG (CdSe2ML_menthylTG). The initial CdSe2ML_OA nanoplatelets were uniform in shape and size and rolled into nanoscrolls due to the effect of spontaneous folding [20,44]. The average lateral size of the scrolled NPLs was about 70 nm. After ligand exchange, the rolled nanoplatelets unrolled to form flat platelets, and the average lateral size of the NPLs was preserved. The unrolling of the rolled nanoplatelets after the addition of menthylTG can be attributed to the larger steric volume of the menthylTG ligand compared to the OA ligand, which hinders spontaneous folding. Also, we confirmed the crystal structure of the atomically thin CdSe nanoplatelets by X-ray diffraction. As grown, CdSe NPLs with an OA ligand have zinc blende crystal structure with an orientation of [001] (Figure S5) and one extra cadmium plane, consistent with reference [20]. After attaching the menthylTG ligand, X-ray diffraction showed that the resulting NPLs retained zinc blende crystal structure. Note that the [220] reflection is shifted to higher angles and broadened due to the effect of the addition of the thiolate group, which results in a compressive deformation of the core and is consistent with reference [20].

### 3.2. Analysis of Chiroptical Properties of NPLs with Chiral Ligand

**Figure 4:**
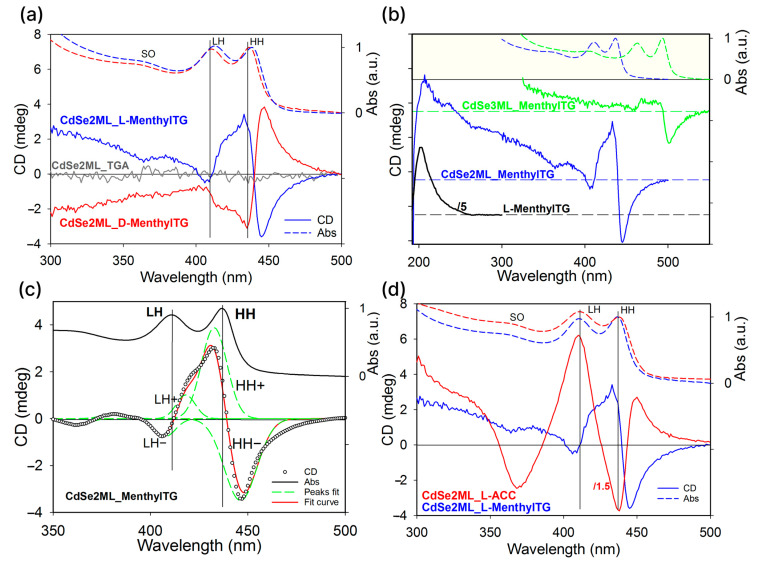
(**a**) CD spectra of 2 ML CdSe NPLs coated with L-(−)-menthyl thioglycolate (blue line), D-(+)-menthyl thioglycolate (red line), and achiral TGA ligands (grey line). (**b**) Comparison of CD spectra of CdSe3ML_L-MenthyTG (green line) and CdSe2ML_L-MenthyTG (blue line) samples emphasizing the effect of thickness. The CD spectrum of free L-MenthyTG (black line) is shown for comparison. (**c**) Decomposition of the CD spectrum of 2 ML CdSe NPLs coated with L-(−)-menthyl thioglycolate into the sum of Lorentz lines. Empty circles are experimental data, dashed green lines are Lorentz profiles, and the red line is the sum of Lorentz profiles. (**d**) Comparison of CD spectra of 2 ML CdSe NPLs coated with L-(−)-menthyl thioglycolate (blue line) and L-acetylcysteine (red line). All panels show the corresponding absorption spectra at the top.

The chiral ligand menthylTG induced chirality in CdSe NPLs after binding to the basal planes, which was reflected by the preferential absorption of left- or right-polarized light by the L- and D-stereoisomers of the NPLs. The chiroptical properties of the NPL samples coated with chiral ligands and the induction of circular dichroism (CD) in the exciton system were analyzed using circular dichroism spectroscopy. Figure 4a shows typical CD spectra of 2ML CdSe NPLs coated with L-(−)-menthyl thioglycolate (blue line) and D-(+)-menthyl thioglycolate (red line). The CD spectra exhibit distinct alternating bands in the spectral range of 300–500 nm, whose positions correlate with the exciton bands in the absorption spectra, indicating a clear Cotton effect in the exciton system. The most intense band of the CD spectra corresponds to the HH exciton and has a negative sign for the L-(−) stereoisomer and a positive sign for the D-(+) one. The change of the L-(−) stereoisomer of the ligand to the D-(+) stereoisomer leads to a mirror reflection of the spectrum, preserving the spectral position and the value of ellipticity. At the same time, the 2 ML CdSe NPL sample coated with an achiral TGA ligand shows no CD signal (gray line). These observations prove the direct effect of the ligand’s chirality on the induced exciton chirality.

Next, we analyze the results for the L-(−)-stereoisomer in more detail. Circular dichroism was also observed for the thicker 3 ML CdSe NPL samples coated with L-(−)-menthyl thioglycolate, similarly to the 2 ML CdSe NPLs, as shown in Figure 4b. Increasing the thickness results in a spectral shift of the exciton absorption bands to longer wavelengths and a simultaneous shift of the CD bands so that, for the 3 ML CdSe NPLs, the CD bands also correlate with the exciton absorption bands. The signs and character of the bands in the CD spectrum for the CdSe3ML_L-MenthyTG sample qualitatively coincide with those of the CdSe2ML_L-MenthyTG, but it is clear that the amplitude of the CD bands decreases sharply with the increasing thickness of the semiconductor core. This is a clear indication of a strong attenuation of the chirality induction effect with an increasing thickness of the NPLs, which is consistent with reference [20]. The maximum value of the dissymmetry g-factor is achieved for the HH exciton in the case of the thinnest 2 ML NPLs and is 2.5 × 10^−4^. For comparison, Figure 4b and Figure S3 show the CD spectrum of free L-(−)-menthyl thioglycolate. As can be seen, the CD band for the free ligand is observed deep in the UV region with a maximum near 210 nm, practically coinciding with the absorption band and corresponding to a positive Cotton effect. It is obvious that the chirality of NPLs is not determined by the manifestation of chiral ligands themselves, but is due to the effect induced by them in the semiconductor core.

As follows from Figure 4a,b, each exciton band in the absorption spectrum corresponds to a change in the sign of the CD bands in the circular dichroism spectrum. For example, the lowest-energy CD band, corresponding to the HH exciton, has a negative sign, which then changes to a band with a positive sign at higher energies. The exciton maximum of the HH exciton in the absorption spectrum approximately corresponds to the zero value in the CD spectrum. The LH exciton also correlates with the features in the CD bands, but its ellipticity is significantly smaller than that of the HH exciton. We decomposed the intense region in the CD spectrum corresponding to the HH and LH excitons into the sum of the negative and positive Lorentz lines, e.g., HH+ and HH− components for the HH exciton and LH+ and LH− components for the LH exciton (Figure 4c), which satisfactorily describes the resulting CD spectrum. The behavior of the CD spectrum for CdSe NPLs with the L-(−)-menthyl thioglycolate ligand is qualitatively different from that of previously studied CdSe NPLs with thiolate ligands of cysteine derivatives (e.g., [20,32]) and carboxylate ligands (e.g., [26]). For comparison, we exchanged the synthesized 2 ML CdSe NPLs with the N-acetyl-L-Cysteine ligand. A comparison of the CD spectra for the N-acetyl-L-Cysteine and L-menthyl thioglycolate ligands is shown in Figure 4d. For the sample with the N-acetyl-L-Cysteine ligand, the lower-energy CD band (HH exciton) is positive for the HH exciton, whereas, for the one with the L-menthyl thioglycolate ligand, it is negative. Moreover, for the N-acetyl-L-Cysteine ligand, the most intense CD band corresponds to the LH exciton, whereas, for the L-menthyl thioglycolate ligand, it corresponds to an HH exciton. A possible reason for the different signs of the observed effects for HH and LH excitons may be the difference in the signs of the spin splitting for the corresponding HH and LH bands.

This behavior of induced chirality should be attributed to the orientation of molecular dipoles of the stereocenter and the exciton, as suggested in [13,17,20,21,32]. For CdSe NPLs, the exciton has a transition dipole in the nanoplatelet plane for the HH exciton, whereas, for the LH exciton, the transition dipole components lie both in the nanoplatelet plane and outside of the plane [45,46]. Changing the sign of the CD when replacing the L-menthyl thioglycolate ligand with the N-acetyl-L-Cysteine one changes the orientation of the molecular dipole of the stereocenter. The contribution in the case of the N-acetyl-L-Cysteine ligand has its stereocenter near the Cd-S bond, whereas, for menthylTG, this will be the 2S stereocenter of the menthol fragment closest to the basal plane. At the same time, it can be inferred from [26] that the maximum signal for the HH exciton in the case of menthylTG may correspond to a strong connection between the molecular dipole oriented parallel to the NPL plane and the HH exciton component in the plane.

### 3.3. Modeling of Crystal Structure by the Density Functional Method and Analysis by the MCD Method

**Figure 5:**
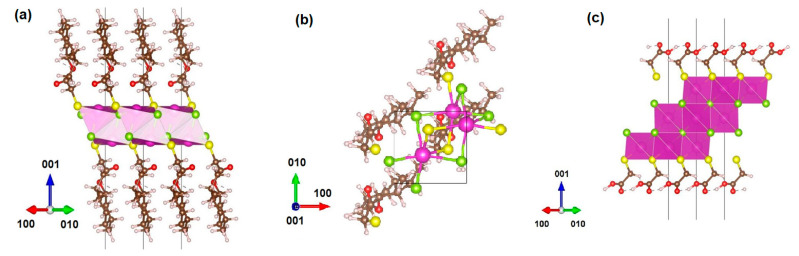
DFT models of the crystal structure of 2 ML CdSe NPLs coated with (**a**) L-(−)-menthyl thioglycolate, in a [110] orientation, (**b**) L-(−)-menthyl thioglycolate, in a [001] orientation, and (**c**) thioglycolic acid, in [110] orientation. Atoms are marked: Cd—green, Se—purple, S—yellow, O—red.

Our experimental study of the chiroptical properties of CdSe NPLs was supplemented by modeling their crystal structure and ligand coordination using the density functional theory (DFT). We used the formula for the composition of atomically thin CdSe NPLs proposed by us earlier [20,39,40] as Cd_n+1_Se_n_L_2_, where L- is the carboxylate ligand RCOO^−^ of oleic acid. Then, taking into account the coordination of the L-menthyl thioglycolate ligand solely by the thiolate group, based on the FTIR data, we can write the composition of the NPLs after the ligand exchange as Cd_n+1_Se_n_(S-R’)_2_, where R’ is the organic fragment of menthol ether. The result of modeling the crystal structure of 2 ML CdSe NPLs coated with L-(−)-menthyl thioglycolate in the [110] and [001] orientations is shown in Figure 5a,b. For comparison, a fragment of the crystal structure of 2 ML CdSe NPLs coated with TGA is shown in Figure 5c.

An analysis of the structure of 2 ML CdSe NPLs coated with L-(−)-menthyl thioglycolate shows a significant distortion of its crystal structure caused by a fairly sterically large ligand. There is a significant in-plane tension (about 30%) of the inorganic core of the nanoplatelet. The L-(−)-menthyl thioglycolate ligand is oriented normally to the plane of the nanoplatelet, and the planes of the cyclohexane fragments are oriented along the [110] direction. The local environment of the central Cd atom in the nanoplatelet is highly distorted: its local environment is represented by four Se atoms from two adjacent Se layers (two atoms at 2.776 Å and two atoms 3.154 Å) and two S atoms from ligands at 2.695 Å. Thus, the perturbation produced by the ligand is localized not only at the surface, but also penetrates deep into the inorganic core. The large perturbation produced by the L-(−)-menthyl thioglycolate ligand results in a strong torsion distortion of CdSe_4_ tetrahedra of the inorganic CdSe core. At the same time, a significantly smaller distortion is observed for the crystal structure of the 2 ML CdSe NPLs coated with the thioglycolic acid ligand. In this case, the local tetrahedral environment of the central Cd atom demonstrates only a minor distortion: four Se atoms in the first shell are nearly at the same distance (two atoms at 2.603 Å and two atoms at 2.606 Å), whereas the surface S atoms are at 4.697 Å. The shifts of the Se atoms in the CdSe4 tetrahedra are such that they do not produce the torsion distortion of the tetrahedron, which is consistent with the achiral nature of the TGA ligand.

**Figure 6:**
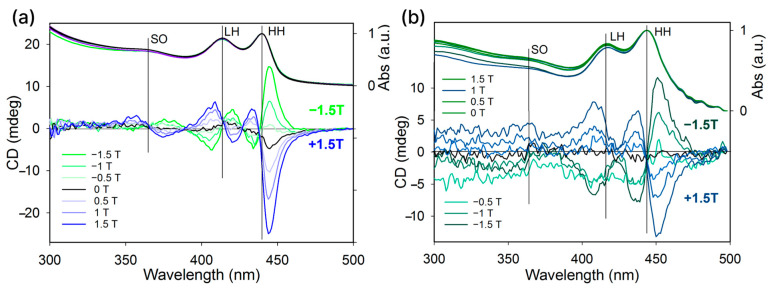
MCD spectra of 2 ML CdSe NPL samples coated with (**a**) L-(−)-menthyl thioglycolate and (**b**) TGA ligands. The spectra were recorded from NPL dispersions in (**a**) THF and (**b**) methanol. The measurements were performed in magnetic fields up to ±1.5 T. The spectra were recorded with an interval of 0.5 T.

To obtain additional information, we performed magnetic circular dichroism (MCD) measurements on 2 ML CdSe NPLs coated with L-(−)-menthyl thioglycolate and TGA ligands, as shown in Figure 6. The 2 ML CdSe NPLs coated with L-(−)-menthyl thioglycolate exhibited distinct alternating MCD bands, clearly corresponding to HH and LH excitons in absorption. In total, four intense bands were observed, corresponding to HH and LH excitons. Both the HH exciton and LH exciton corresponded to a pair of alternating bands, where the low-energy band had a positive sign in a positive magnetic field, and the band with higher energy had a negative sign. The minima of the spectra corresponded to the exciton maxima. The inversion of the sign of the magnetic field lead to a mirror image of the spectra. The maximum value of the MCD ellipticity is 15 mdeg for a magnetic field of ±1.5 T at an optical density of 1 and an optical length of 1 cm. At the same time, the behavior of the MCD spectra of the 2 ML CdSe NPLs coated with TGA ligands was different. The spectra contained clear alternating MCD bands corresponding to HH and LH excitons, but only three intense bands corresponding to HH and LH excitons were observed. A pair of alternating bands corresponding to the LH exciton merged into a single band. The maximum value of MCD ellipticity is 10 mdeg for a magnetic field of ±1.5 T at an optical density of 1. Such different shapes of the MCD spectra may indicate different distortions of the crystal structure induced by the L-(−)-menthyl thioglycolate and TGA ligands, consistent with the simulation results.

## 4. Conclusions

We have demonstrated the induction of chirality in atomically thin 2 and 3 ML CdSe nanoplatelets induced by menthol derivatives containing multiple stereocenters. We designed a novel chiral ligand based on an ester of menthol and thioglycolic acid, L-(−)/D-(+)-menthyl thioglycolate, which contains a sulfhydryl group as an anchor for attachment to the nanoplatelet surface and a menthol fragment that induces chirality in the semiconductor cores of NPLs. It was shown that L-(−)/D-(+)-menthyl thioglycolate readily attaches to the basal cadmium planes of the nanoplatelets by replacing the native oleic acid. This allows obtaining chiral CdSe NPLs with a non-polar surface (due to the hydrocarbon cyclohexane moiety of menthol) and an affinity for non-polar solvents, which distinguishes them from the well-known chiral CdSe NPLs with cysteine-based ligands that have a polar surface and which are soluble in polar solvents. The addition of the ligand to the basal planes of the nanoplatelets is accompanied by a decrease in the energy of the exciton bands, which results from a greater delocalization of the exciton due to the addition of two atomic planes of sulfur atoms. We observed a pronounced circular dichroism of CdSe NPLs coated with L-(−)- and D-(+)-menthyl thioglycolate, demonstrating narrow alternating CD bands which clearly correspond to HH and LH absorption bands. In comparison with the N-acetyl-L-Cysteine ligand, the CD bands of CdSe NPLs coated with L-(−)-menthyl thioglycolate had the opposite sign. This effect should be addressed to the different orientations of molecular dipoles in the molecules of these ligands relative to the exciton in the nanoplatelet. The most intense CD bands corresponded to the lower-energy HH exciton. The magnitude of the circular dichroism sharply decreased when the NPL thickness was increased from 2 to 3 ML, and a maximal dissymmetry g-factor up to 2.5 × 10^−4^ was found for the HH exciton for the 2 ML NPLs. First-principles modeling of the crystal structure revealed a strong distortion of the semiconductor core, which was confirmed by magnetic circular dichroism measurements. We believe that our work may be of interest for the design of new chiral semiconductor nanostructures and the understanding of their chiroptical properties. The proposed new chiral menthyl thioglycolate ligand can be used to induce chirality in different two-dimensional materials with basal cationic surfaces. Moreover, novel effects can be expected due to the sterically bulky structure and multiple stereocenters of the resulting nanostructure, which can be expected to be used as a chiral auxiliary in asymmetric photocatalysis.

## Data Availability

The original contributions presented in the study are included in the article/Supplementary Materials, further inquiries can be directed to the corresponding author.

## Supplementary Materials

The following supporting information can be downloaded at: https://www.mdpi.com/article/10.3390/nano14231921/s1. Figure S1. H-NMR spectrum of Menthyl thioglycolate, dissolved in d-chloroform. Figure S2. H-NMR spectrum of menthol. Figure S3. H-NMR spectrum of thioglycolic acid. Figure S4. Typical absorbance spectra of CdSe2ML_MenthylTG and CdSe2ML_TGA. Figure S5. X-ray diffraction patterns collected from as-synthesized CdSe2ML_OA and CdSe2ML_MenthylTG. Figure S6. CD spectrum and absorption spectrum of the CdSe3ML_MenthylTG. References [47,48] are cited in the Supplementary Materials.
