# Supplementary material for: Menthol-Induced Chirality in Semiconductor Nanostructures: Chiroptical Properties of Atomically Thin 2D CdSe Nanoplatelets Capped with Enantiomeric L-(−)/D-(+)-Menthyl Thioglycolates
Source: Nanomaterials (Basel). 2024 Nov 28;14(23):1921. doi: 10.3390/nano14231921 (PMC11643598; doi:10.3390/nano14231921)
Supplement: Supplementary file 1 [file nanomaterials-14-01921-s001.zip › nanomaterials-3331340-supplementary.pdf]

Supporting Information:

Menthol-induced chirality in semiconductor nanoplatelets: chiroptical properties of atomically thin 2D CdSe nanostruc-tures capped with enantiomeric L-(−)/D-(+)-Menthyl Thioglycolates

*Maria Yu. Skrypnik[1], Daria A. Kurtina[1], Sofia P. Karamysheva[2], Evgeniia A. Stepanidenko[2], Irina S. Vasil'eva[3], Shuai Chang[4], Alexander I. Lebedev[5], and Roman B. Vasiliev [1,6,]**

1 Department of Chemistry, Lomonosov Moscow State University, 119991 Moscow, Russia; skrypnikmy@my.msu.ru (M.Yu.S) dashutakarlova@mail.ru (D.A.K.)
2 PhysNano Department, ITMO University, Kronverksky pr.49, 197101, St Petersburg, Russia; spkaramysheva@itmo.ru (S.P.K.); eastepanidenko@itmo.ru (E.A.S)
3 A. N. Bach Institute of Biochemistry, Research Center of Biotechnology of the Russian Academy of Sciences, Leninsky Ave. 33, Bld. 2, 119071 Moscow, Russia; ir-vas@yandex.ru
4 Faculty of Materials Science, Shenzhen MSU-BIT University, Shenzhen 518115, China; schang@smbu.edu.cn
5 Department of Physics, Lomonosov Moscow State University, 119991 Moscow, Russia; swan@scon155.phys.msu.ru
6 Department of Materials Science, Lomonosov Moscow State University, 119991 Moscow, Russia
* Correspondence: romvas@inorg.chem.msu.ru

Contents

Figure S1. H-NMR spectrum of Menthyl thioglycolate, dissolved in d-chloroform.

Figure S2. H-NMR spectrum of menthol.

Figure S3. H-NMR spectrum of thioglycolic acid.

Figure S4. Typical absorbance spectra of CdSe2ML_MenthylTG and CdSe2ML_TGA.

Figure S5. X-ray diffraction patterns collected from as-synthesized CdSe2ML_OA and CdSe2ML_MenthylTG.

Figure S6. CD spectrum and absorption spectrum of the CdSe3ML_MenthylTG.

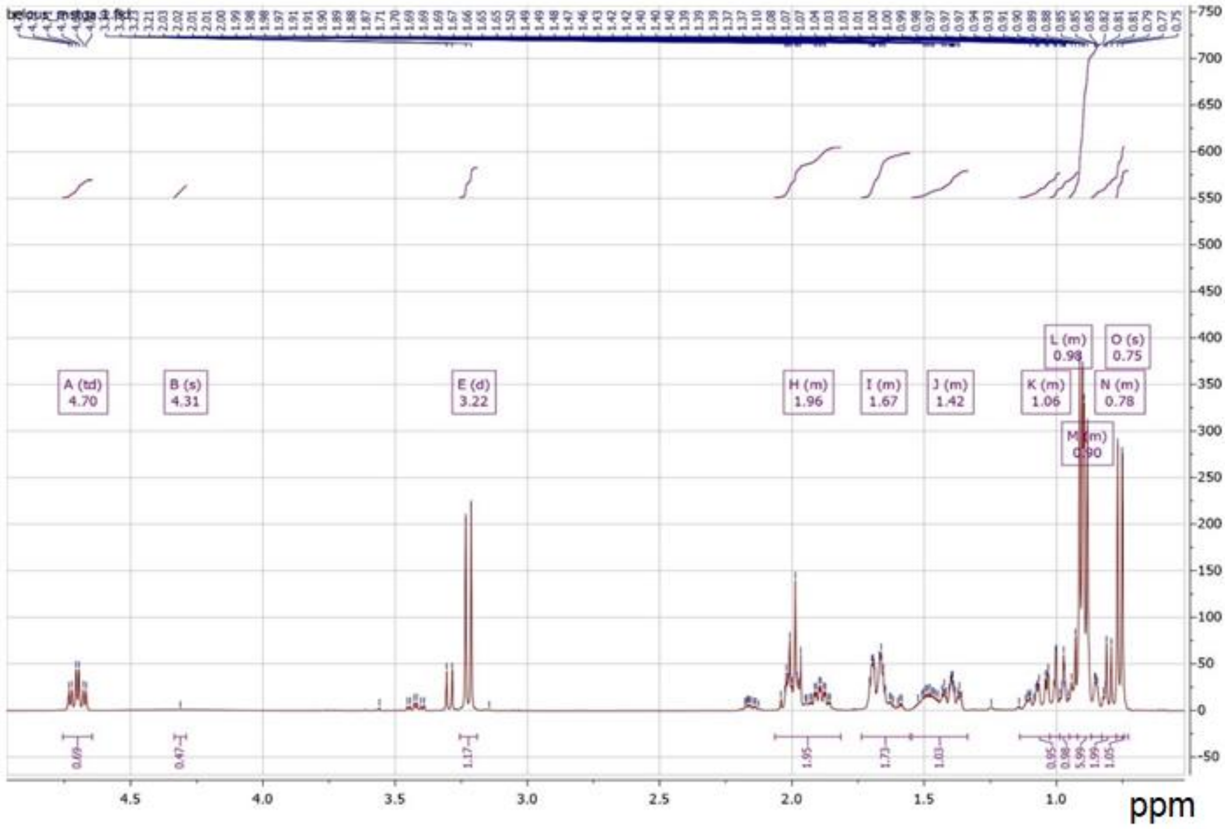

Figure S1. H-NMR spectrum of Menthyl thioglycolate, dissolved in d-chloroform. ^{1}**H NMR:** (600 MHz, Chloroform-d) δ 0.75 (d, 3H, CH3), 0,9 (d, J=3,18 Hz, 3H, CH3), 0.92 (d, J=4.22 Hz, 3H, CH3), 0,92 – 1,09 (m, 3H), 1.34 – 1.55 (m, 2H), 1.55 – 1.74 (m, 2H), 1,82 - 2,06 (m, 3H), 3.21 and 3.23 (d, J = 8.3 Hz, 2s, 2H, CH2S), 4.70 (td, J = 10.9, 4.4 Hz, 1H, CHOCO).

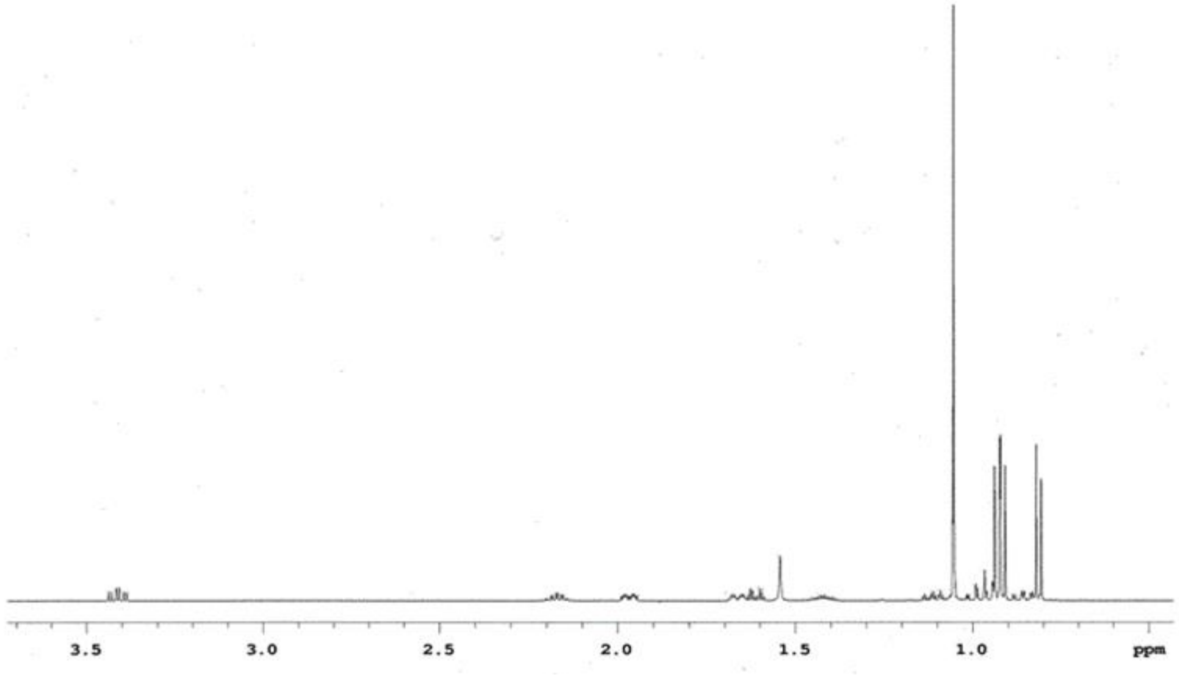

Figure S2. H-NMR spectrum of menthol [1]. ^{1}**H NMR:** (500 MHz, CDCl3) δ = 0.82 (d,3H), 0.92 (d, 6H), 1.05 (m, 1H), 1.63 (m, 1H), 3.41 (m, 1H) ppm.

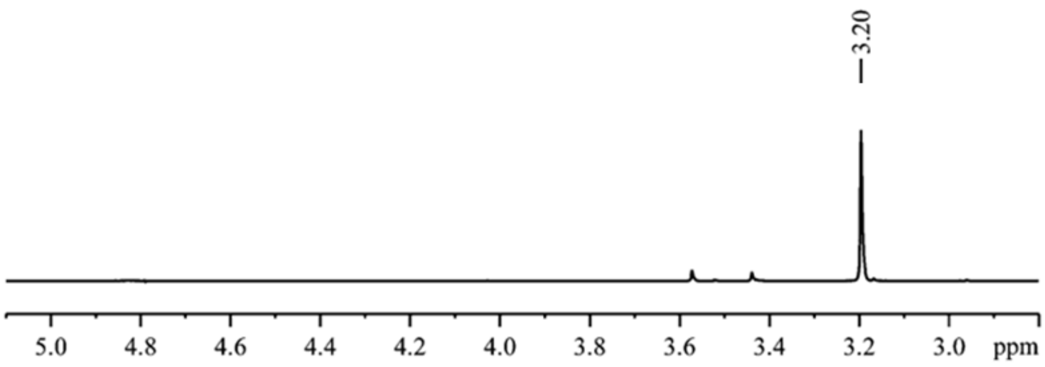

Figure S3. H-NMR spectrum of thioglycolic acid [2].

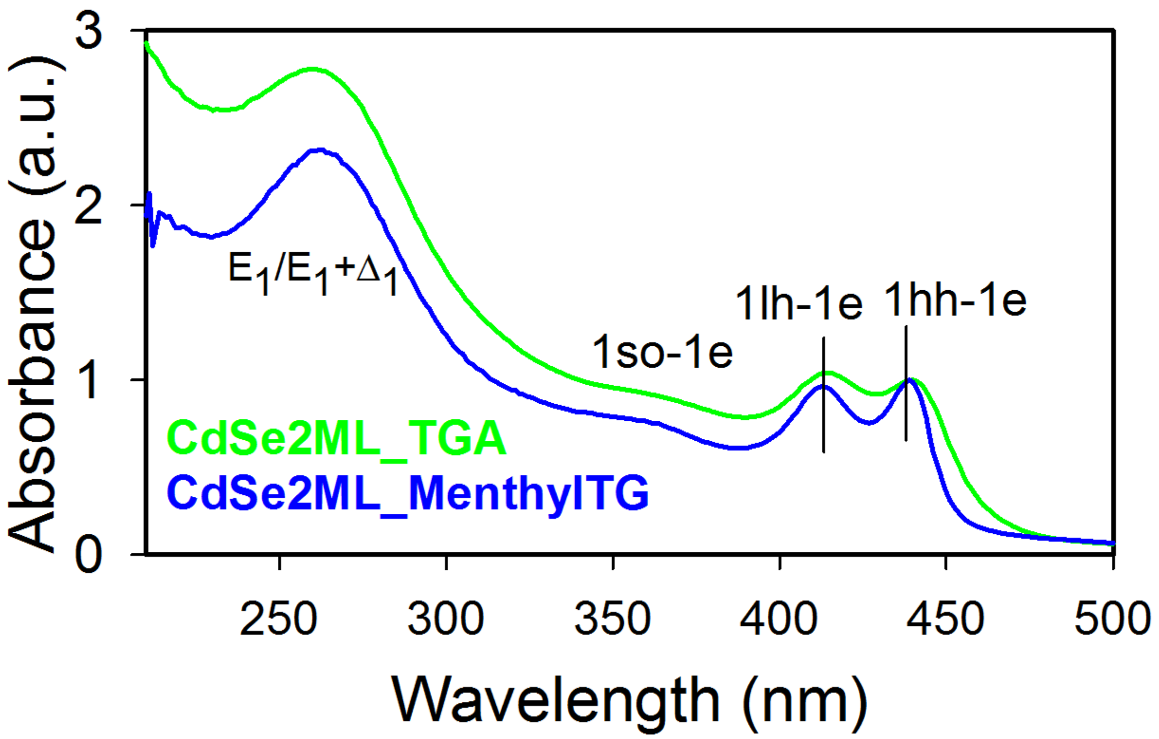

Figure S4. Typical absorbance spectra of CdSe2ML NPLs after ligand exchange with MenthylTG (CdSe2ML_MenthylTG, blue line) and thioglycolic acid (CdSe2ML_TGA, green line) ligands.

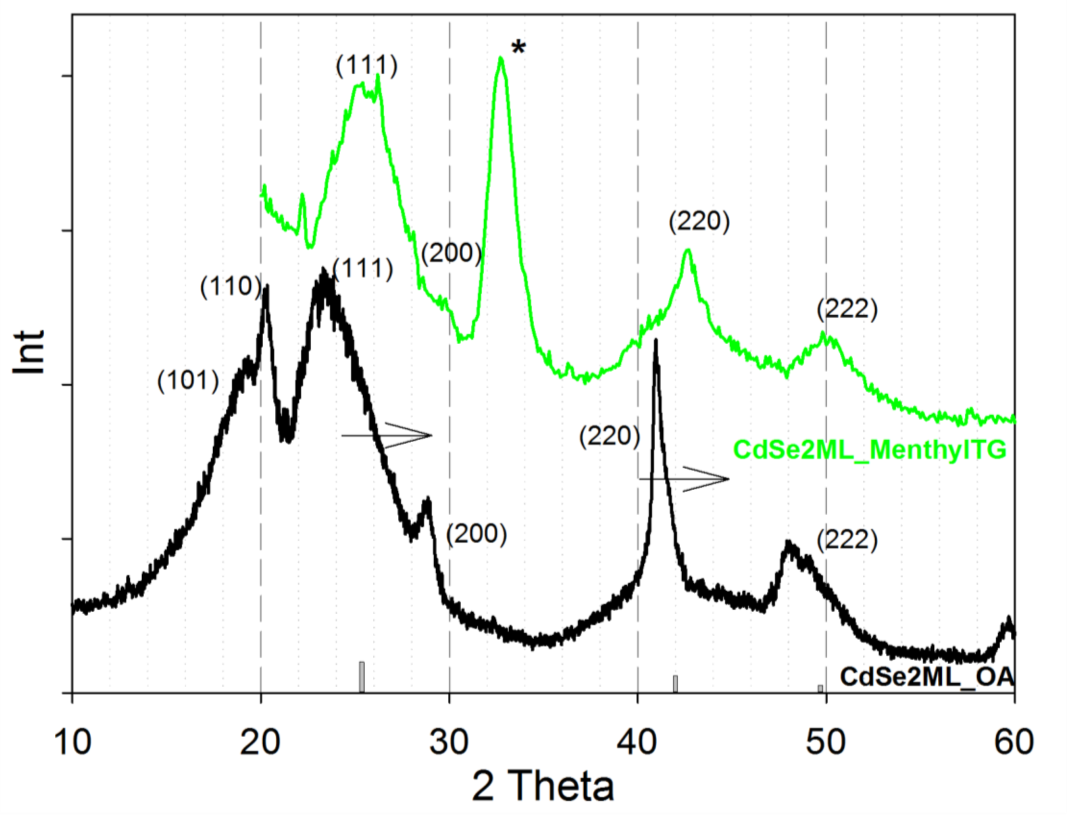

Figure S5. X-ray diffraction patterns collected from as-synthesized CdSe2ML_OA (black solid line) and CdSe2ML_MenthylTG (green solid line). Standard diffraction peaks for the zinc blende crystal structure of CdSe (black vertical lines, JCPDS card no. 19-191) are shown at the bottom. An asterisk marks a reflex originating from a silicon substrate.

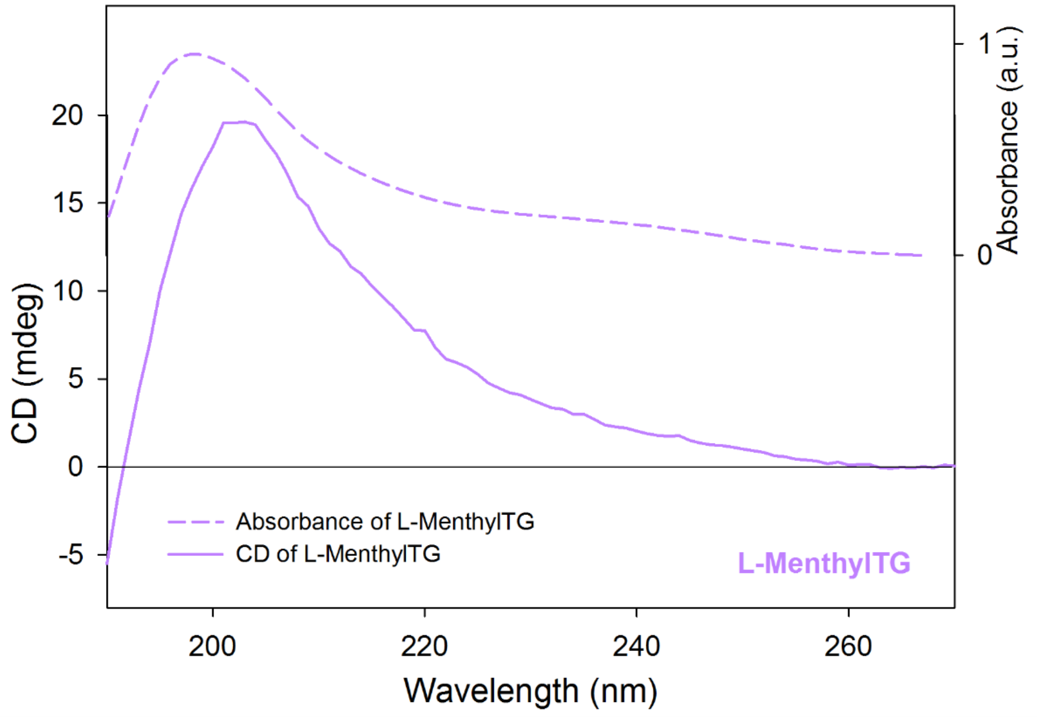

Figure S5. UV part of the CD spectrum (solid line) and absorption spectrum (dashed line) of the free ligand L-(−)-Methyl thioglycolate dissolved in methanol.

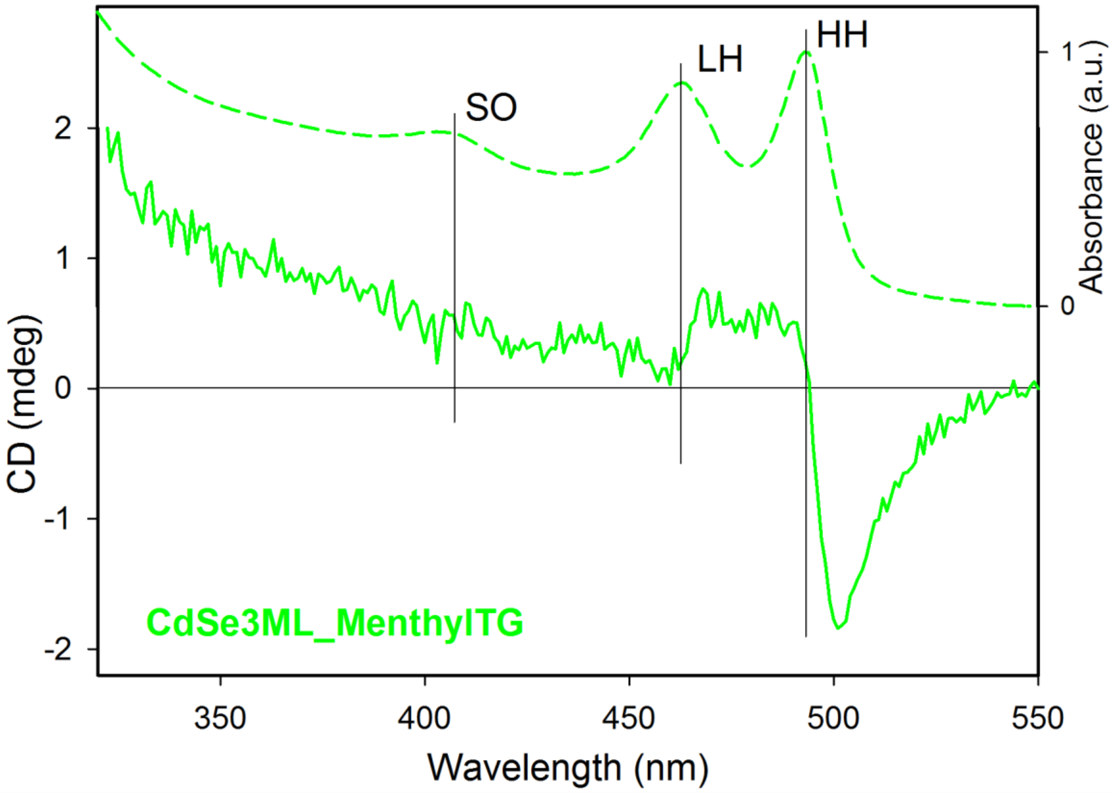

Figure S6. CD spectrum (solid line) and absorption spectrum (dashed line) of the 3 ML thick NPLs CdSe3ML_MenthylTG.

References.

1. González-Calderón, D.; Benítez-Puebla, L.J.; González-González, C.A.; Assad-Hernández, S.; Fuentes-Benítez, A.; Cuevas-Yáñez, E.; Corona-Becerril, D.; González-Romero, C. Selective deprotection of TBDMS alkyl ethers in the presence of TIPS or TBDPS phenyl ethers by catalytic CuSO4·5H2O in methanol. *Tetrahedron Letters* **2013**, 54, 5130-5132. DOI: 10.1016/j.tetlet.2013.07.074
2. Schuetze, B.; Mayer, C.; Loza, K.; Gocyla, M.; Heggenc, M.; Epple, M. Conjugation of thiol-terminated molecules to ultrasmall 2 nm-gold nanoparticles leads to remarkably complex 1H-NMR spectra. *J. Mater. Chem. B* **2016**, 4, 2179-2189. DOI: 10.1039/C5TB02443A
